# Superior ophthalmic vein thrombosis associated with severe facial trauma: a case report

**DOI:** 10.1186/s13256-015-0737-y

**Published:** 2015-10-30

**Authors:** Momoko Mishima, Tetsuya Yumoto, Hiroaki Hashimoto, Takao Yasuhara, Atsuyoshi Iida, Kohei Tsukahara, Keiji Sato, Toyomu Ugawa, Fumio Otsuka, Yoshihito Ujike

**Affiliations:** 1 Center for Graduate Medical Education, Okayama University Hospital, 2-5-1 Shikata-cho, Kita-ku, Okayama-shi, Okayama 700-8558 Japan; 2 Advanced Emergency and Critical Care Medical Center, Okayama University Hospital, Okayama, 700-8558 Japan; 3 Department of Neurological Surgery, Okayama University Graduate School of Medicine, Dentistry and Pharmaceutical Sciences, Okayama, 700-8558 Japan

**Keywords:** Carotid cavernous fistula, Facial trauma, Superior ophthalmic vein thrombosis

## Abstract

**Introduction:**

Superior ophthalmic vein thrombosis is a rare entity, but is associated with significant morbidities. We describe a case in which superior ophthalmic vein thrombosis occurred shortly after severe facial trauma.

**Case presentation:**

A 77-year-old Japanese man was transferred to our tertiary hospital after a motor vehicle accident. Le Fort III facial bone fractures and a minor cerebral contusion were detected. Follow-up computed tomography scans detected dilatation of his left superior ophthalmic vein on day 3 and thrombosis on day 12; however, no causative carotid cavernous fistula was observed. As he did not present with any symptoms other than slight conjunctival congestion, a conservative management strategy was adopted along with anticoagulant therapy against deep venous thrombosis. The superior ophthalmic vein thrombosis resolved spontaneously and the conjunctival congestion also improved.

**Conclusions:**

Superior ophthalmic vein thrombosis should be taken into consideration and managed properly after severe facial injuries, and further investigation of its cause is necessary to prevent morbidities.

## Introduction

Superior ophthalmic vein (SOV) thrombosis, which is extremely rare, can have various causes such as orbital infection and tumors, or traumatic or spontaneous carotid cavernous fistulas (CCF) [1–6]. SOV thrombosis can present with proptosis, chemosis, conjunctival congestion, and/or visual disturbance, and can be detected with contrast-enhanced computed tomography (CT) or magnetic resonance imaging (MRI) [4, 6, 7]. Appropriate intervention and management strategies based on the underlying disease and the patient’s clinical symptoms are required; otherwise, severe complications can occur [3]. We report a case in which SOV thrombosis occurred after severe facial injuries.

## Case presentation

**Fig. 1:**
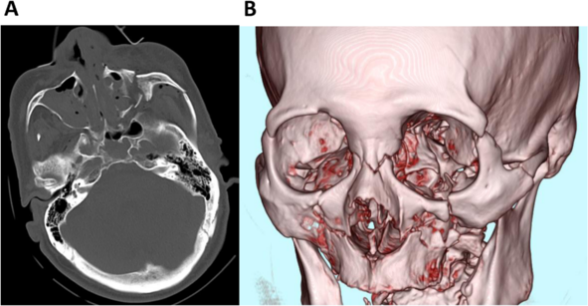
Craniofacial computed tomography scan obtained on arrival. **a** Bilateral fractures of the maxillary sinus wall and fluid collection were detected. Complex fractures were observed on the patient’s left side. **b** A three-dimensional computed tomography scan showed fractures extending through the nose, along the floor and medial and lateral walls of the orbit, and across the left zygomatic bone

A 77-year-old Japanese man, who was hit by a midsize truck while driving a motorcycle, was transported by ambulance to our tertiary hospital after sustaining severe injuries to his face. On arrival, he presented with bleeding from his nose and swelling of an eyelid, which prevented his ophthalmologic condition from being properly evaluated; however, he did not complain of visual impairment. The ophthalmologist examined him on day one, which revealed that the intraocular pressure was within normal range bilaterally with no relative afferent pupillary defect. The pupillary light reflex was absent on the patient’s right side, which was suggestive of traumatic mydriasis, which resolved in a week. His vital signs were as follows: Glasgow Coma Scale 14, respiratory rate 22 breaths/minute, pulse rate: 77 beats/minute, and blood pressure: 203/95 mmHg. He was diagnosed with: a frontal bone fracture; a minor epidural hematoma; a cerebral contusion; Le Fort III fractures of his nose, bilateral orbital walls, and left zygomatic arch; multiple rib fractures; and a fracture of his left ulna based on X-rays and a CT scan (Fig. 1).

**Fig. 2:**
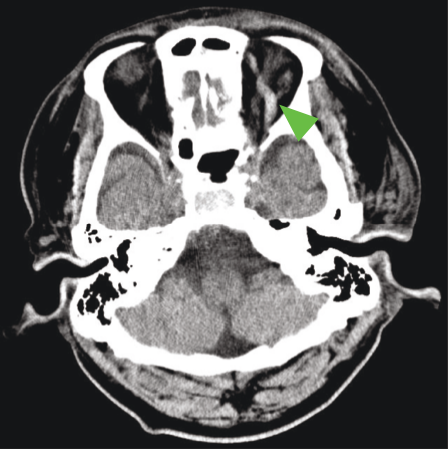
Follow-up plain computed tomography scan conducted on day 3 to screen for head injuries. The *yellow-green arrowhead* indicates the dilated left superior ophthalmic vein (to 4.8 mm)

**Fig. 3:**
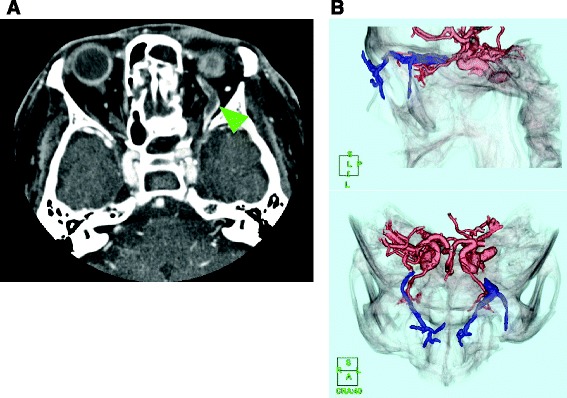
Contrast-enhanced computed tomography scan with angiography performed on day 12 to screen for superior ophthalmic vein engorgement. **a** The *yellow-green arrowhead* shows the dilated left superior ophthalmic vein (to 5.9 mm) together with a non-enhanced mass, which was indicative of thrombosis. **b** Computed tomography angiography did not detect any obvious arteriovenous fistulas or abnormal cavernous sinus enhancement

**Fig. 4:**
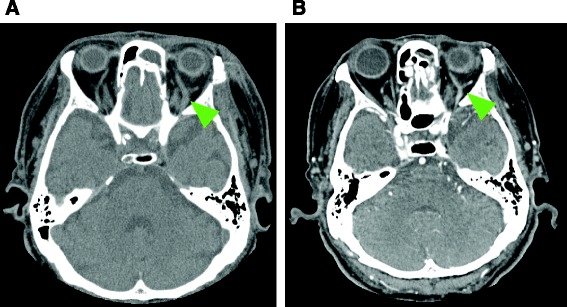
Follow-up computed tomography scan of the superior ophthalmic vein thrombosis. A plain scan was conducted on day 17 (**a**) and a contrast-enhanced scan was performed on day 17 (**b**). **a** The *yellow-green arrowhead* shows that the dilatation of the superior ophthalmic vein had improved (to 4.3 mm). **b** The *yellow-green arrowhead* shows that the dilatation of the superior ophthalmic vein had improved further (to 3.3 mm) and that the thrombosis had almost been resolved

He was intubated and mechanically ventilated for 11 days to protect his airway from bleeding from his facial injuries. Although he had no traumatic brain injuries (TBI) that required surgical treatment, cerebrospinal fluid rhinorrhea was treated via a lumber drain, which resolved within 10 days. On day 3, a follow-up plain CT scan for TBI revealed mild dilatation of his left SOV, which was suggestive of a CCF or SOV thrombosis resulting from insufficient venous drainage (Fig. 2). As he did not exhibit exophthalmos, audible pulsatile bruits, extraocular muscle disturbances, or visual impairment (except for slight hyperemia of the left conjunctiva), careful follow-up and observation were continued during the period of sedation and mechanical ventilation. Unfractionated heparin was started on day 8 for a deep venous thrombosis extending from his left external iliac vein to his femoral vein. On day 12, although a contrast-enhanced CT scan showed expansion and thrombosis of his left SOV and these findings did not exhibit any obvious connection with the internal carotid artery or the cavernous sinus, which was suggestive of a CCF (Fig. 3), he was conservatively managed due to the asymptomatic nature of his condition. On day 14, his complex facial fractures were surgically repaired to correct cosmetic defects and masticatory problems. The dilation of his left SOV and slight hyperemia of the conjunctiva improved within 2 weeks (Fig. 4). Although he possessed binocular vision, he complained of a slight visual disturbance associated with subluxation of his right lens when he was transferred to another hospital to undergo rehabilitation on the 31st hospital day, at which time he was receiving warfarin for a residual deep venous thrombosis. He did not have any complaints about his left eye at 4 months after the injury.

## Discussion

SOV thrombosis is an extremely rare entity resulting from orbital congestion, such as that caused by infectious diseases, for example: paranasal sinusitis, orbital cellulitis, or septic cavernous sinus thrombosis; cavernous sinus, orbital, or skull base tumors; arteriovenous malformations; or cavernous sinus thrombosis [1–5]. The structural changes associated with aging are also related to venous thrombosis [8]. Intrasinusal venous pressure affects the cerebral venous system; its hypertension decreases cerebral blood flow and produces intracerebral venous congestion [8, 9]. Regardless of whether they are traumatic in origin, CCF can cause SOV thrombosis due to retrograde drainage from the cavernous sinus into the SOV, but the frequency of this condition is unknown [6]. CCF-related thrombosis of the SOV can sometimes occur following the embolization of a CCF, which is called paradoxical worsening [10, 11]. In the present case, the causative mechanism of the patient’s SOV thrombosis was unknown; however, several etiologies were considered. First, the occurrence of a traumatic intraorbital arteriovenous fistula; that is, an indirect or low flow CCF was considered because the patient was asymptomatic except for slight conjunctival congestion, and no cavernous sinus enhancement was detected on CT angiography. Second, direct occlusion of the SOV due to a sphenoid bone fracture or hematoma, blocking the flow of blood into the cavernous sinus, was another possible mechanism, but no such fractures or hematomas were seen in this case. Furthermore, orbital compartment syndrome and orbital infection were incompatible with the SOV thrombosis seen in the present case based on the patient’s symptoms and clinical course.

SOV thrombosis can manifest as proptosis, chemosis, conjunctival congestion, diplopia, and visual impairment [4, 7]. In addition to these findings, the symptoms of CCF include headache, cephalic bruit, and elevated intraocular pressure [12]. The present patient did not exhibit any symptoms other than slight conjunctival congestion associated with SOV thrombosis, which was explained by the use of collateral venous drainage routes, such as the inferior ophthalmic vein and facial vein [13].

SOV thrombosis is usually identified by contrast-enhanced CT or MRI followed by an assessment of the patient’s clinical symptoms [6]. In our patient, follow-up CT for TBI detected SOV engorgement and thrombosis. The normal maximal diameter of the SOV was reported to be 3.5 mm [3, 14]. Although angiography might have established the cause of the patient’s SOV thrombosis, it was not performed because the patient’s symptoms indicated that his condition was not urgent, and a follow-up CT scan revealed that his SOV had resolved spontaneously.

The optimal treatment for SOV thrombosis depends on the etiology and clinical symptoms of the condition. Endovascular treatment via an arterial or venous approach is preferred for cases involving CCF [15]. Indirect or low flow CCF will often resolve spontaneously [16]. Infection-related SOV thromboses require immediate surgical drainage and antibiotic administration to prevent ophthalmologic complications [3]. Although the utility of anticoagulant therapy for infection-related SOV thrombosis is disputed, we treated our patient with heparin followed by warfarin for a deep venous thrombosis, which seemed to be effective against his SOV thrombosis [3, 6, 17].

## Conclusions

We described a case of SOV thrombosis associated with severe facial injuries, which improved spontaneously. Careful observation of such cases for ophthalmologic symptoms and further investigations of the cause of the SOV thrombosis are necessary to prevent morbidities.

## Consent

Written informed consent was obtained from the patient for publication of this case report and accompanying images. A copy of the written consent is available for review by the Editor-in-Chief of this journal.

## Abbreviations

CCF: Carotid cavernous fistulas
CT: Computed tomography
MRI: Magnetic resonance imaging
SOV: Superior ophthalmic vein
TBI: Traumatic brain injuries
